# Genomic and Metabolomic Landscape of Right-Sided and Left-Sided Colorectal Cancer: Potential Preventive Biomarkers

**DOI:** 10.3390/cells11030527

**Published:** 2022-02-03

**Authors:** Ming-Wei Su, Chung-Ke Chang, Chien-Wei Lin, Hou-Wei Chu, Tsen-Ni Tsai, Wei-Chih Su, Yen-Cheng Chen, Tsung-Kun Chang, Ching-Wen Huang, Hsiang-Lin Tsai, Chang-Chieh Wu, Huang-Chi Chou, Bei-Hao Shiu, Jaw-Yuan Wang

**Affiliations:** 1 Institute of Biomedical Sciences, Academia Sinica, Taipei 115, Taiwan; wei@ibms.sinica.edu.tw (M.-W.S.); chungke@ibms.sinica.edu.tw (C.-K.C.); rover1023@ibms.sinica.edu.tw (C.-W.L.); howei@ibms.sinica.edu.tw (H.-W.C.); 2 Division of Colorectal Surgery, Department of Surgery, Kaohsiung Medical University Hospital, Kaohsiung Medical University, Kaohsiung 807, Taiwan; jennytsai1978@yahoo.com.tw (T.-N.T.); lake0126@yahoo.com.tw (W.-C.S.); googoogi05@gmail.com (Y.-C.C.); tsungkunchang@gmail.com (T.-K.C.); baseball5824@yahoo.com.tw (C.-W.H.); chunpin870132@yahoo.com.tw (H.-L.T.); 3 Graduate Institute of Clinical Medicine, College of Medicine, Kaohsiung Medical University, Kaohsiung 807, Taiwan; 4 Department of Surgery, Faculty of Medicine, College of Medicine, Kaohsiung Medical University, Kaohsiung 807, Taiwan; 5 Department of Surgery, Tri-Service General Hospital Keelung Branch, National Defense Medical Center, Keelung 20042, Taiwan; doc20276@gmail.com; 6 School of Medicine, Chung Shan Medical University, Taichung 402306, Taiwan; hcjy341@gmail.com (H.-C.C.); shiubeihao@gmail.com (B.-H.S.); 7 Division of Colon and Rectal Surgery, Department of Surgery, Chung Shan Medical University Hospital, Taichung 402306, Taiwan; 8 Center for Cancer Research, Kaohsiung Medical University, Kaohsiung 807, Taiwan; 9 Center for Liquid Biopsy and Cohort Research, Kaohsiung Medical University, Kaohsiung 807, Taiwan; 10 Pingtung Hospital, Ministry of Health and Welfare, Pingtung 900, Taiwan

**Keywords:** colorectal cancer, whole exome sequence, polygenic risk score, metabolomic profiling

## Abstract

Colorectal cancer (CRC) is the third most common cancer worldwide. The incidence and mortality rates of CRC are significantly higher in Taiwan than in other developed countries. Genes involved in CRC tumorigenesis differ depending on whether the tumor occurs on the left or right side of the colon, and genomic analysis is a keystone in the study and treatment of CRC subtypes. However, few studies have focused on the genetic landscape of Taiwanese patients with CRC. This study comprehensively analyzed the genomes of 141 Taiwanese patients with CRC through whole-exome sequencing. Significant genomic differences related to the site of CRC development were observed. Blood metabolomic profiling and polygenic risk score analysis were performed to identify potential biomarkers for the early identification and prevention of CRC in the Taiwanese population. Our findings provide vital clues for establishing population-specific treatments and health policies for CRC prevention in Taiwan.

## 1. Introduction

Colorectal cancer (CRC) is the third most common cancer worldwide and has a high mortality rate at advanced stages. Compared to other developed countries, the incidence and mortality rates of CRC are higher in Taiwan, where CRC has been the most common type of malignancy and the third leading cause of cancer-related deaths since 1996. According to data from the Taiwanese Ministry of Health and Welfare, CRC incidence in Taiwan increased from 32.38 to 66.32 per 100,000 individuals between the years 2000 and 2017. Mortality rates also increased from 20.6 per 100,000 individuals in 2009 to 27.3 per 100,000 individuals in 2019 [1]. As with most cancers, CRC is not a monolithic disease with a single cause but rather involves several possible genetic mechanisms. Mutations of genes involved in signaling pathways, including APC (the Wnt pathway), KRAS (the EGFR/MAPK pathway), PIK3CA (the PI3K/Akt pathway) and SMAD-2/3/4 (the TGF-beta pathway), are commonly associated with various CRC subtypes [2]. In addition, mutations in TP53 and DNA mismatch repair genes play key roles in certain types of CRC. These different mechanisms are not completely independent of each other, and multiple mechanisms may or may not be involved in different cases of CRC, thereby adding another layer of molecular complexity to the disease. Because of these characteristics, developing general all-purpose treatments for CRC can be difficult; a precision medicine approach might be more suitable. However, few studies have examined the genetic landscape of Taiwanese CRC patients, and this lack of data may impede the development of such an approach.

The colon is commonly divided into the left and right sides. The left side includes the region between the splenic flexure and the upper anal canal; the right side refers to the proximal colon, including the cecum, ascending colon, and transverse colon. The embryological origins and biological characteristics differ considerably between these two sides. As a consequence, the epidemiological, pathological, cytogenetic, and molecular features of left-sided CRC (LCRC) differ substantially from those of right-sided CRC (RCRC) [3]. Hu et al. found that RCRC contained more aggressive molecular markers than in LCRC, including the overexpression of oncogenic micro-RNAs such as miR-10b, higher microsatellite instability (MSI), and several BRAF and KRAS mutations [4]. In addition, the prognoses of RCRC and LCRC differ significantly [5,6,7,8,9,10]. Prognostic differences have been mainly observed in patients with stage III CRC and metastatic CRC [9,10]. Likewise, targeted treatments for metastatic LCRC and RCRC are also different. For example, anti-epidermal growth factor receptor (anti-EGFR) treatment is recommended for patients with wild-type RAS LCRC, whereas anti-vascular endothelial growth factor (anti-VEGF) treatment is preferred for patients with metastatic wild-type RAS RCRC [11,12,13]. Recognizing the differences between LCRC and RCRC is crucial for understanding CRC progression and designing suitable therapeutic strategies.

Because CRC has a strong genetic basis, a polygenic risk score (PRS) may facilitate the development of strategies for the early detection and prevention of CRC. Law et al. developed a PRS based on 79 single-nucleotide polymorphisms (SNPs) that were identified through genome-wide association studies in the European population [14]. They found that individuals in the top 1% of the PRS scale had a considerably higher risk of developing CRC (2.6-fold) compared with the general population. Metabolomic approaches have also been increasingly applied to understand carcinogenesis and aid the development of therapeutic methods. In general, the metabolic phenotypes of cancer cells are different from those of normal cells, and alterations in blood metabolite levels are observed in most cancer types, including CRC [15]. Studies have extensively explored the metabolic phenotypes of many cancer types but not of CRC. However, because of the diversity in the genetic backgrounds of individuals of different ethnicities, the applicability of the PRS and metabolomics to Asian cohorts remains debatable. The effect of tumor sidedness, i.e., LCRC or RCRC, on both PRS and blood metabolomic changes is also not clear.

In this study, we explored the genomic landscape of tumor sidedness in Taiwanese patients with CRC and examined differences between Taiwanese and Caucasian patients. The PRS for CRC developed by Law et al. was applicable to the Taiwanese population, with younger CRC patients exhibiting higher PRSs. LCRC and RCRC were successfully differentiated in blood metabolomic profiling, with sarcosine constituting a presumed biomarker. Our study provides several new findings relevant to the development of precision medicine treatments for CRC, particularly for the Asian population.

## 2. Materials and Methods

### 2.1. Clinical Specimens

Blood samples, as well as CRC tissues and adjacent normal tissues, were prospectively collected from 141 patients with CRC from Kaohsiung Medical University, Chung-Ho Memorial Hospital (KMUH), Tri-Service General Hospital, and Chung Shan Medical University Hospital between 2016 and 2020. An additional 326 patients were later recruited for the polygenic risk score (PRS) study. The patients’ clinical information, comprising their age, sex, histology, cancer stage, and primary tumor location, was collected. TNM staging was performed according to the criteria established by the American Joint Commission on Cancer and the Union for International Cancer Control [16]. We included treatment-naive patients who were newly diagnosed with CRC and who were undergoing primary surgery for CRC. We excluded patients who had received neoadjuvant chemotherapy. The Research Ethics Committee of KMUH approved the study protocol (KMUHIRB-G(II)-20200029), and all patients provided written informed consent to participate.

### 2.2. Genomic Data Acquisition

Whole-exome sequencing of both tumor and adjacent normal tissues was conducted by Genomics, Inc. (Taipei, Taiwan). Genomic DNA was isolated from frozen tissues using a taco™ Total DNA Extraction Kit (GeneReach Biotechnology Corp., Taichung, Taiwan). The ratios of the absorbances at 260 nm and 280 nm (A260/280) of the isolated DNA samples were measured on an Agilent Bioanalyzer 2100 (Agilent, Santa Clara, CA, USA), and samples with A260/280 ratios between 1.7 and 2.0 were deemed to be of sufficient quality for exome sequencing. An Agilent SureSelectXT Human All Exon V6 + Cosmic Target Enrichment System was used for exome capture. The concentrations of all libraries were quantified with an Invitrogen Qubit 2.0 fluorometer (Thermo Fisher Scientific, Waltham, MA, USA). Paired-end sequencing was performed using an Illumina NovaSeq 6000 sequencer (Illumina, San Diego, CA, USA). The minimum sequence depth of the target regions was 300×. Whole-genome sequences and genotyping data of healthy controls were directly obtained from the Taiwan Biobank [17,18].

### 2.3. Detection of Somatic Mutations in CRC Tumor Samples

DNA sequencing reads were passed through the Trim Galore! wrapper script (https://www.bioinformatics.babraham.ac.uk/projects/trim_galore/, accessed on 29 January 2022) for quality control. Reads that passed the quality control process were then aligned against the GRCh38 version of the human reference genome with BWA-MEM v0.7.15 [19]. The aligned sequences were processed using the GATK suite [20]. FixMateInformation was used to verify and correct the alignment as necessary. Subsequently, duplicate reads were categorized and marked using the program markDuplicate, which also allowed a base quality score recalibration. Somatic mutations in the cleaned sequences were then determined using MuTect2 [21]. Somatic mutations identified at this stage still contained a considerable number of false signals. Thus, we used the panel of normals (PON) approach to filter out these false signals. We used 515 whole-genome sequences from the Taiwan Biobank to establish our PON in the noise detection mode of MuTect2, a somatic variant caller. Somatic mutations originally determined using MuTect2 were compared against the PON, and mutations present in at least two samples of the PON were discarded. Finally, errors in read orientation and sample contamination were removed using the Learn Read Orientation Model and Calculate Contamination in the GATK Suite, respectively.

### 2.4. Microsatellite Instability (MSI) Detection and Tumor Mutational Burden (TMB)

MSI is often caused by DNA mismatch repair and is associated with several cancer types, including CRC. The samples were classified into MSI-high and MSI-stable groups using the binary predictor described in [22]. TMB is a measure of the number of somatic mutations within a tumor and is presented as the number of somatic mutations per coding sequence length [23].

### 2.5. Metabolomic Profiling

Serum samples obtained from patients with CRC were prepared according to established protocols [24]. Raw nuclear magnetic resonance (NMR) free induction decay data were acquired using the cpmgpr1d pulse sequence on a Bruker Avance 800-MHz spectrometer (Bruker, Karlsruhe, Germany). Line broadening (0.3 Hz) was applied to all free induction decay data prior to the Fourier transform in Bruker Topspin v3.5pl7. The spectral phase was manually corrected within the program. The water region between 4 and 5 ppm was removed from all spectra. Baseline correction, resonance signal alignment, and intelligent binning were performed using the Rnmr1D package within the R statistical environment v3.5 [25]. Putative assignment of the sarcosine signal was based on the Human Metabolome Database [26]. Supervised partial least squares discriminant analysis (PLS-DA) was performed using the R package mixOmics v6.1.1 to differentiate between LCRC and RCRC metabolomic profiles [27].

### 2.6. Polygenic Risk Analysis

Genotypes of 467 patients with CRC and 1000 controls without a history of cancer were obtained from the Taiwan Biobank. The weighted PRS was calculated with PLINK v1.9 [28] based on the weights of 79 SNPs shown in [14]. All relevant SNPs were included in the calculations, regardless of their imputation quality. SNPs with missing data were excluded.

## 3. Results

### 3.1. Genomic Landscape of the Taiwanese CRC Cohort

**Figure 1:**
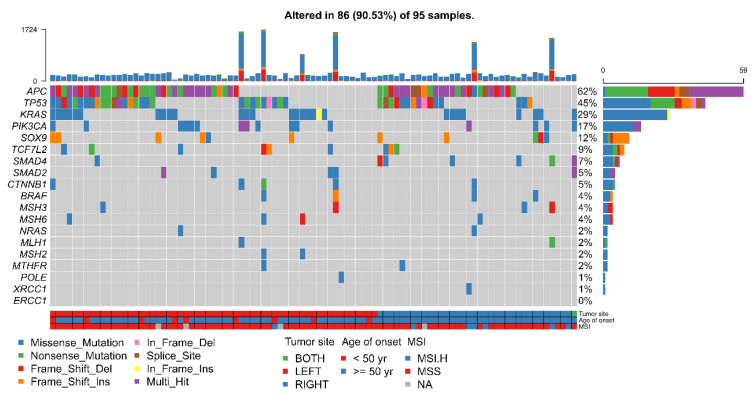
Genomic landscape of the Taiwanese CRC cohort.

**Figure 2:**
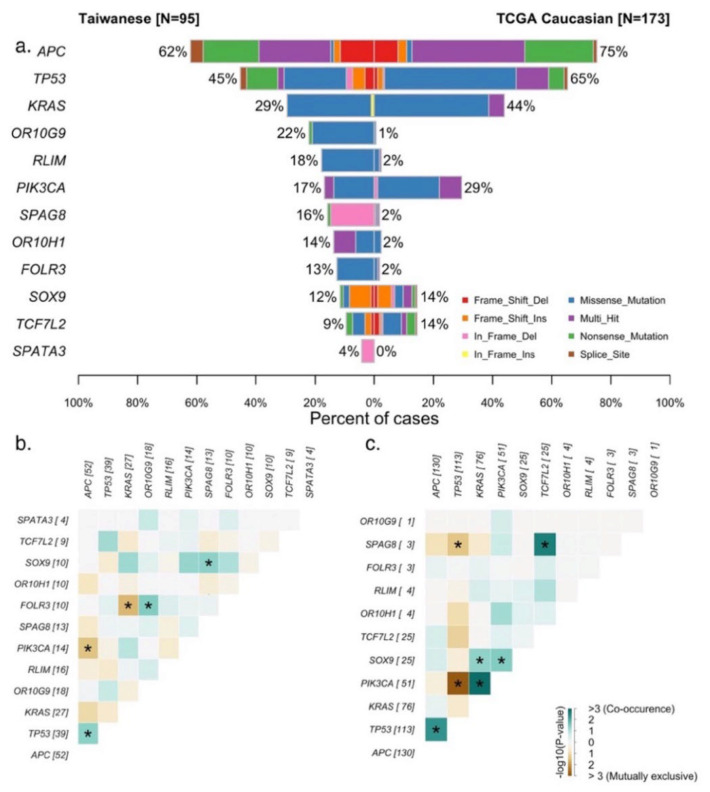
Genetic comparison of the Taiwanese CRC cohort with the Caucasian cohort in the TCGA. (**a**) Co-bar plots of the mutational frequencies of genes in the two cohorts. (**b**) Somatic gene interactions in the Taiwanese cohort. (**c**) Somatic gene interactions in the Caucasian cohort.

To prevent potential bias resulting from presurgical treatment, we selected 95 treatment-naive patients undergoing primary surgery for colorectal adenocarcinoma. Figure 1 presents the findings in the Taiwanese CRC cohort on genes known to play crucial roles in CRC carcinogenesis. Driver mutations of the Taiwanese cohort were located in FOLR3, KRAS, OR10G9, OR10H1, SPATA3, TP53, APC, SPAG8, SOX9, RLIM, PIK3CA, and TCF7L2 (q ≤ 0.01). Although many of these genes, such as APC, KRAS, TP53, and PIK3CA, are canonical CRC oncogenes, several distinct features potentially specific to the Taiwanese cohort were identified. Mutations in APC were observed in 62% of the samples, which is lower than the APC mutation rate of 75% (Chi-square test, *p*-value = 0.03) reported for Caucasians in The Cancer Genome Atlas (TCGA) (Figure 2a). Moreover, the RAS/BRAF mutation rate of the Taiwanese cohort was lower than that of the Caucasian cohort in the TCGA (Chi-square test, *p*-value = 0.0005). However, our Taiwanese cohort exhibited a higher rate of MSI than the Caucasian cohort (12/141 vs. 7/173, respectively; Chi-square test, *p*-value = 0.01), as shown in Table 1. RCRC patients had a later age of onset and a higher rate of poorly differentiated tumors compared to LCRC patients. There was no difference in the gender composition, BMI distribution, TMB, or cancer stage between LCRC and RCRC patients. Notably, 9 (~10%) out of 95 patients in the Taiwanese cohort did not exhibit any canonical mutation; of these 9 patients, 6 had LCRC, whereas 3 had RCRC. A mutual exclusivity analysis of the major driver mutations revealed that APC and TP53 mutations co-occurred most frequently in the Taiwanese cohort (Figure 2a). Mutually exclusive mutations include APC vs. PIK3CA and KRAS vs. FOLR3. However, the TCGA Caucasian cohort exhibited a different pattern, with PIK3CA and KRAS mutations having the most frequent co-occurrence, whereas PIK3CA mutations are mutually exclusive with TP53 mutations (Figure 2b,c). These results suggest the involvement of different driver genes and alternative carcinogenic pathways between the Caucasian and Taiwanese cohorts.

### 3.2. Comparison of Genetic Features between LCRC and RCRC in the Taiwanese and Caucasian Cohorts

In the Taiwanese cohort, PIK3CA and KRAS mutations were more commonly observed in LCRC, whereas APC mutations were more commonly observed in RCRC (Table 2). TP53 mutations, on the other hand, were observed at similar frequencies between LCRC and RCRC in the Taiwanese cohort. The opposite was true for the TCGA Caucasian cohort, with APC mutations occurring more often in LCRC, whereas KRAS and PIK3CA mutations were more commonly observed in RCRC. TP53 mutations were also more often observed in LCRC compared to RCRC in Caucasians (Chi-square test, *p*-value = 0.19). When comparing the same TNM stages between different cohorts, Caucasians generally had higher mutation rates for all genes, with the exception of KRAS and PIK3CA in advanced-stage (TNM III + IV) LCRC. Detailed examination revealed that KRAS mutation rates of Taiwanese are especially low in early-stage (TNM I + II) RCRC.

The differences between LCRC and RCRC in the Taiwanese cohort were not only limited to canonical genes but also extended to those involved in DNA stability. Among the 12 MSI-high patients, 11 had RCRC, in agreement with previous studies [29]. Likewise, the overall TMB was higher in RCRC compared to LCRC (Student’s t-test, *p*-value > 0.05). In addition, SMAD2 and SMAD4 mutations, which are associated with chromosomal stability, were observed more frequently in LCRC and RCRC, respectively (Chi-square test, *p*-value = 0.78). Our results suggest that when discussing tumor sidedness, the genetic differences between Taiwanese and Caucasians may reside in the canonical CRC genes instead of genes involved in the maintenance of DNA stability.

### 3.3. Metabolomic Profiling of LCRC and RCRC

**Figure 3:**
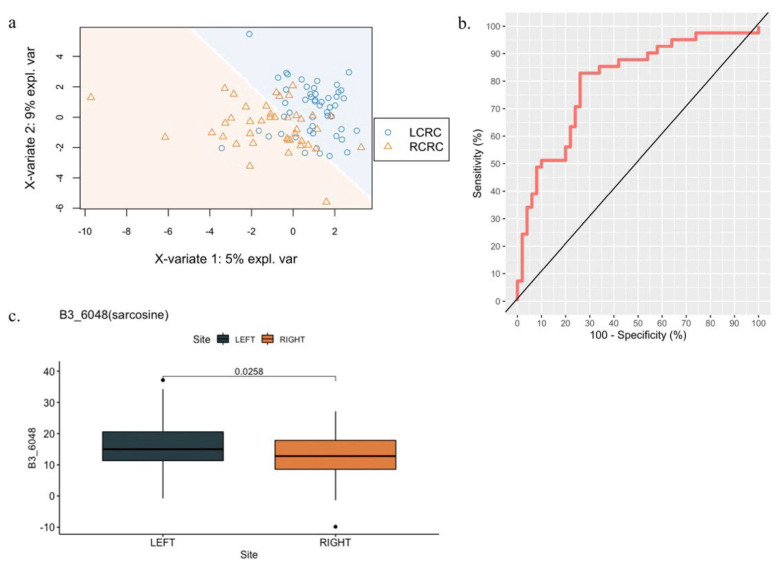
Metabolomic profiling of LCRC and RCRC: (**a**) PLS-DA plot based on all spectral signals; (**b**) response operator curve differentiating between LCRC and RCRC based on all spectral signals; (**c**) box plot of the putative signal from sarcosine at 3.605 ppm.

We employed a chemometric approach for the blood metabolomic profiling of patients with LCRC and RCRC. The binned intensities of each NMR resonance signal across all spectra were used for the PLS-DA, which is presented in Figure 3a. A moderate separation was observed between LCRC and RCRC, and the area under the receiver operating characteristic curve using the full spectral data was 0.8 (Figure 3b). Among the top five candidates, only the bin at approximately 3.605 ppm had significant signal amplitude and was investigated further (see the Supplementary Figure S1). We observed a significant difference in the 3.605-ppm signal intensities between LCRC and RCRC samples (Figure 3c). By comparing these with the Human Metabolome Database (https://hmdb.ca, accessed on 18 June 2021), the signals most likely originated from sarcosine, which has been implicated in several cancer types.

### 3.4. PRS of Taiwanese Subjects

**Figure 4:**
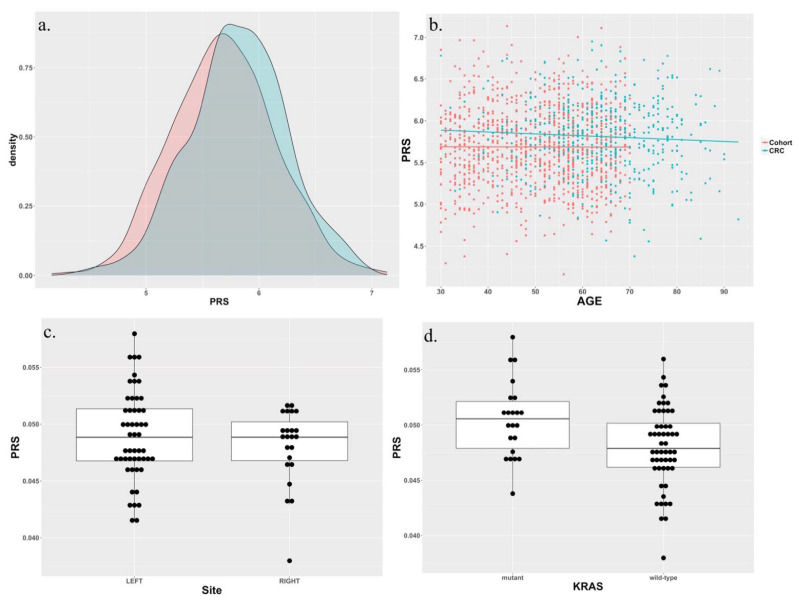
(**a**) PRS values of patients with CRC and controls. (**b**) Correlation of PRS values and the age of onset of CRC. For the 467 CRC patients, the regression line slope is −0.0022 (*p*-value = 0.2). For the 1000 control samples, the regression line slope is −1 × 10^−4^ (*p*-value = 0.94). (**c**) PRS differences between LCRC and RCRC (Student’s t-test; *p*-value = 0.31). (**d**) PRS differences between CRC patients with wild-type KRAS and mutant KRAS (Student’s t-test; *p*-value = 0.008).

We examined the PRS values of 467 patients with CRC (see the Supplementary Table S1) and compared them to the PRS values of 1000 controls without cancer history. Data for the controls were obtained from the Taiwan Biobank. Using the SNPs and weights identified by Law et al., we found that patients with CRC had higher PRS values than did the controls (Student’s t-test, *p*-value = 7.09 × 10^−7^ (Figure 4a), suggesting that the PRS values originally developed using European data may be applicable to the Taiwanese population. The association between the PRS values and early CRC onset was expected because a higher PRS value reflects a higher risk of CRC development early in life (Figure 4b). This trend was not observed in the control group. We did not detect differences in PRS values between LCRC and RCRC (Figure 4c). On the other hand, KRAS mutations were positively associated with the PRS values (Figure 4d). After adjustments were made for age and gender, patients with PRS values in the top 5% showed a significant CRC odds ratio of 1.82 compared with the remaining patients. Surprisingly, when we applied our data on a PRS based on the East Asian population, no statistical differences were observed between CRC patients and healthy controls in terms of PRS values (Student’s t-test, *p*-value = 0.18) (see Supplementary Figure S2) [30].

## 4. Discussion

To the best of our knowledge, this is one of the few studies on CRC conducted in Asian populations to also leverage information from a population biobank. The genomic landscape of the Taiwanese cohort differed considerably from that of the Caucasian TCGA cohort. The genomic characteristics of LCRC and RCRC in the Taiwanese cohort were also distinct from those in the Caucasian cohort, possibly reflecting ethnic differences in treatment outcomes between patients in the East and the West. For example, the MSI rate of the Taiwanese CRC cohort was only half of that of the Caucasian cohort. The APC mutation rate, one of the most crucial genetic factors in CRC, was lower in the Taiwanese CRC cohort than in the Caucasian cohort. A similar trend was observed in the TP53, KRAS, and PIK3CA mutation rates. Both the MSI and oncogene results were consistent with those of previous studies conducted on Chinese patients with CRC [31,32]. However, TP53 mutations were more dominant than APC mutations in the Chinese cohort, whereas the opposite was true for the Taiwanese cohort. This discrepancy suggests subtle differences in genetic mechanisms involved in CRC pathogenesis between Chinese and Taiwanese patients.

Of particular interest is the comparison between our cohort and a Japanese CRC cohort [33]. The APC and TP53 mutation rates in the Japanese cohort were even higher than those in the Caucasian cohort; however, the prevalence of CRC in both Japan and Western countries was lower than that in Taiwan. These comparative observations suggest a smaller-than-expected contribution of canonical CRC genes to CRC prevalence. Additional genes and non-genetic factors, such as diet and environmental status, may contribute significantly to high CRC prevalence in Asia.

The differences among populations extend to the genomic landscapes of LCRC and RCRC. In the Taiwanese CRC cohort, APC mutations were more common in RCRC, whereas KRAS and PIK3CA mutations were more common in LCRC. Although the TP53 mutation rates were comparable between LCRC and RCRC, the rates were significantly increased in patients with LCRC in the advanced stages (III and IV). In contrast, both APC and TP53 mutations were significantly associated with LCRC in the Japanese cohort. Although the differences may be subtle, they highlight the diversity of genomic features among different Asian cohorts with CRC and indicate the potential need for population-specific profiling and treatment strategies.

Cancer risk is determined by the complex interplay of environmental and genetic factors. Although genes such as TP53, KRAS, and APC play crucial mechanistic roles in CRC pathogenesis, they alone may not be sufficient to correctly estimate CRC risk in different populations. A PRS accounting for a wider set of genes that may not have apparent mechanistic associations with disease has been widely employed as a tool for estimating disease risk, including the risks of several cancer types [34]. Our results demonstrate the advantages of using PRS to estimate cancer risk. Although the PRS used in Figure 4 was not specifically developed for all Asian populations, it reflected differences between the control and CRC groups. Surprisingly, the PRS based on SNPs identified by He et al., which presumably was targeted in the East Asian population, fared worse in differentiating CRC vs. normal groups in our cohort. One possible explanation lies in the different numbers of SNPs used to calculate the PRS; Law et al. identified a total of 79 SNPs, whereas He et al. identified only 19 SNPs. A larger number of SNPs may improve the accuracy of the resulting PRS [35]. Regardless, our results suggest that the PRS based on SNPs identified in one cohort may be applicable to other cohorts with different ethnic distributions.

The unexpected association between PRS and KRAS somatic mutations was notable. Because the PRS was calculated from germline data, this association may indicate that certain genotypes can affect the susceptibility of specific oncogenes to mutations. Because of the importance of KRAS in CRC carcinogenesis, understanding biological mechanisms underlying the association between KRAS and other genes may provide opportunities to develop novel CRC treatments for our population.

The inclusion of metabolomic data complements our genomic findings. Similar to patients with cancer in general, patients with CRC have considerably different serum metabolic profiles from those of controls without cancer [36]. However, metabolomic differences between LCRC and RCRC remain unclear. In this study, the raw chemometric profile of the serum NMR spectra was able to differentiate between LCRC and RCRC (Figure 3). This is particularly notable because we expected that any differences between tumor sidedness would be diluted by the systemic nature of human blood circulation. The higher level of the presumed sarcosine signal observed for LCRC (compared with that noted for RCRC) warrants further investigation. Sarcosine has recently been implicated in the methylation of genes associated with several diseases, including CRC [37,38,39]. In one study, the incubation of prostate cells with sarcosine stimulated DNA methyltransferases (DNMTs), resulting in increases in global DNA methylation [37]. In another study, aberrant DNMT expression was reported to be associated with CRC and was proposed as a potential therapeutic target [40]. The different levels of sarcosine between patients with LCRC and RCRC may indicate discrepancies in the methylation status of the genome.

The differences observed among different subgroups in our cohort highlight the need for the development of personalized CRC interventions, even for individuals within relatively homogeneous populations. Genetic and metabolic differences observed between LCRC and RCRC are particularly compelling because they provide clues to potential mechanisms that can be targeted in different individuals.

This study has several limitations. First, the number of CRC cases is relatively small due to the requirement to collect treatment-naïve participants and may limit the statistical power. Second, the distribution of the number of cases across different cancer stages is not identical between LCRC and RCRC because of the small sample size. Third, because a regional cohort was used, the results may not be generalizable to other cohorts. Further investigations using larger cohorts with finer stratification and preferably different ethnic constitutions are warranted.

## 5. Conclusions

In this study, we examined a Taiwanese CRC cohort and compared its characteristics with cohorts from other countries. We observed significant differences in driver gene mutation rates between the Taiwanese and the Caucasian cohorts. PRS values based on germline gene polymorphisms were higher for CRC cases compared to controls. Blood metabolome results were able to differentiate between LCRC and RCRC. Our data suggests that germline mutations and the blood metabolome can be further developed as both preventive and diagnostic biomarkers for CRC.

## Figures and Tables

**Table 1:** Characteristics of the Taiwanese CRC cohort.

	LCRC	RCRC	Both	Total	*p*-Value
	*n* = 80	*n* = 59	*n* = 2	*n* = 141	
Age, Mean (SD)	63.1 (12.3)	68.5 (10.8)	57.5 (0.7)	65.2 (11.9)	<0.01
Gender, Male (%)	47 (58.8%)	30 (50.8%)	1 (50.0%)	78 (55.3%)	
BMI, Mean (SD)	24.0 (3.9)	23.2 (3.6)	21.2 (0.2)	23.6 (3.8)	
Grade, *n* (%)					<0.01
I Well differentiated	1 (1.2%)	4 (6.8%)	2 (100%)	7 (5.0%)	
II Moderate differentiated	72 (90.0%)	44 (74.6%)	0 (0%)	116 (82.3%)	
III Poorly differentiated	2 (2.5%)	9 (15.3%)	0 (0%)	11 (7.8%)	
Stage, *n* (%)					
I	7 (8.8%)	3 (5.1%)	0 (0%)	10 (7.1%)	
II	23 (28.8%)	21 (35.6%)	1 (50%)	45 (31.9%)	
III	34 (42.5%)	22 (37.3%)	1 (50%)	57 (40.4%)	
IV	11 (13.8%)	4 (6.8%)	0 (0%)	15 (10.6%)	
TMB, Mean (SD)	12.9 (48.2)	21.0 (52.8)	2.66 (0.44)	16.2 (49.8)	
MSI, MSI.H (%)	1 (1.2%)	11 (18.6%)	0 (0%)	12 (8.5%)	<0.01

RCRC, right-sided CRC; LCRC, left-sided CRC; TMB, tumor mutational burden (per megabase); MSI, microsatellite instability. “Both” denotes tumor growth in both left and right sides of the colon.

**Table 2:** Comparison of genetic mutation rates in LCRC and RCRC.

Taiwanese Cohort	LCRC (*n* = 59)			RCRC (*n* = 35)		
TNM stage	I + II (*n* = 26)	III + IV (*n* = 33)	overall	I + II (*n* = 16)	III + IV (*n* = 19)	overall
*APC*	54%	61%	58%	62%	76%	71%
*TP53*	38%	55%	47%	38%	53%	43%
*KRAS*	31%	36%	34%	6%	35%	20%
*PIK3CA*	23%	18%	20%	6%	12%	9%
**TCGA Caucasian**	**LCRC (*n* = 67)**			**RCRC (*n* = 85)**		
TNM stage	I + II (*n* = 34)	III + IV (*n* = 33)		I + II (*n* = 48)	III + IV (*n* = 37)	
*APC*	85%	91%	88%	71%	68%	69%
*TP53*	71%	85%	78%	46%	70%	57%
*KRAS*	44%	24%	34%	52%	54%	53%
*PIK3CA*	27%	6%	16%	38%	43%	40%

LCRC, left-sided CRC; RCRC, right-sided CRC; TCGA, The Cancer Genome Atlas.

## Data Availability

The data presented in this study are available on request from the corresponding author. The data are not publicly available due to privacy or ethical restrictions.

## Supplementary Materials

The following supporting information can be downloaded at: https://www.mdpi.com/article/10.3390/cells11030527/s1, Figure S1: Spectral regions of the top 5 contributors of the PLS-DA loadings plot.title; Figure S2: PRS of patients with CRC and controls based on the East Asian population; Table S1: The clinicohystopathological data of the 467 CRC patients for PRS analysistitle.
