# Supplementary material for: Genomic and Metabolomic Landscape of Right-Sided and Left-Sided Colorectal Cancer: Potential Preventive Biomarkers
Source: Cells. 2022 Feb 3;11(3):527. doi: 10.3390/cells11030527 (PMC8834628; doi:10.3390/cells11030527)
Supplement: Supplementary file 1 [file cells-11-00527-s001.zip › cells-1547872-supplementary.pdf]

Supplementary Material

Supplementary Table S1. The clinicohystopathological data for the 467 CRC patients used for PRS analysis.

	n = 467
Age, Mean (SD)	64.1 (11.7)
Gender, Male (%)	272 (58.2%)
BMI, Mean (SD)	24.4 (4.2)
Grade, n (%)	
I Well differentiated	19 (5.2%)
II Moderate differentiated	320 (87.9%)
III Poorly differentiated	25 (6.9%)
Stage, n (%)	
I	94 (26.1%)
II	106 (29.4%)
III	120 (33.3%)
IV	40 (11.1%)

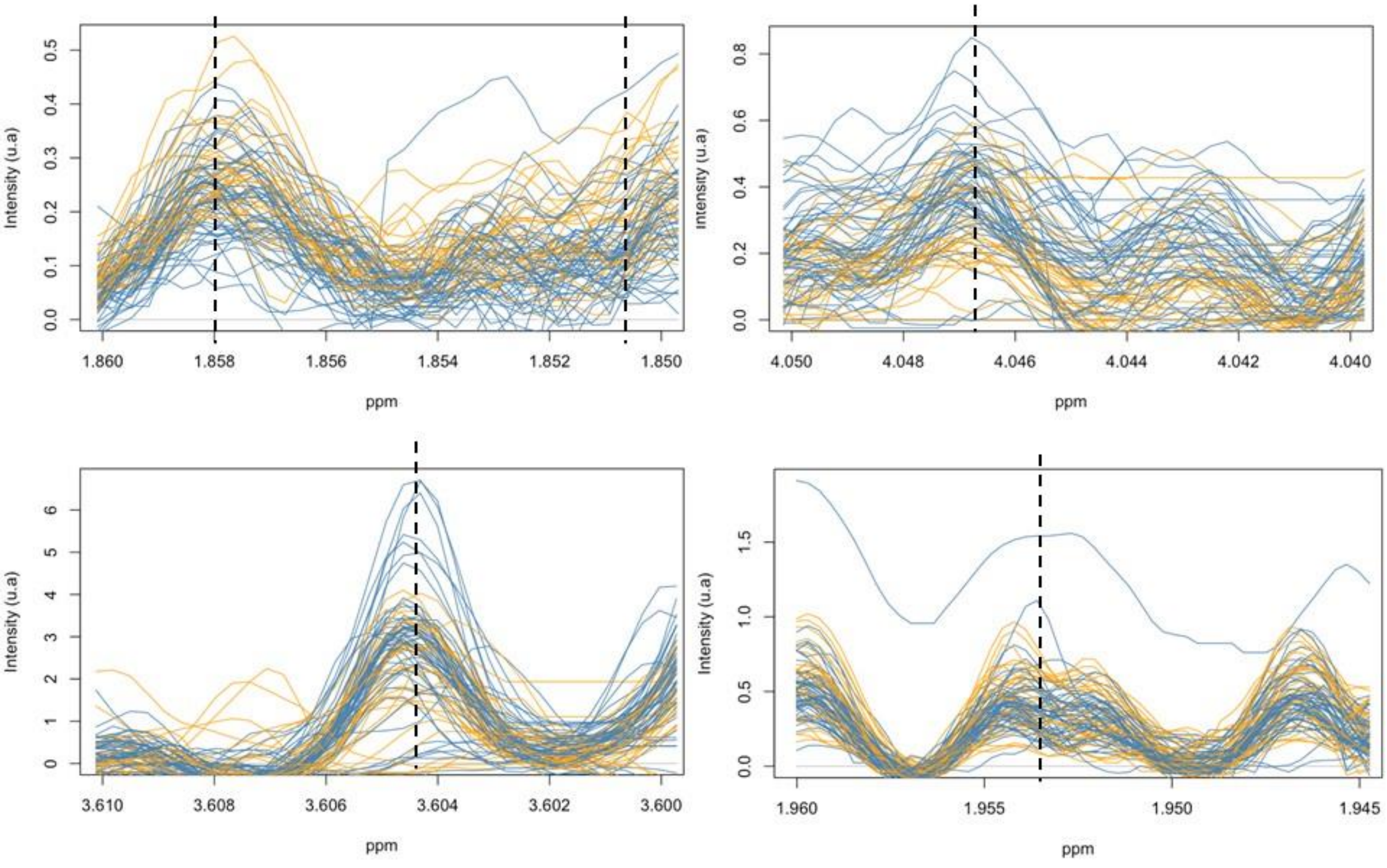

Figure S1. Spectral regions of the top 5 contributors of the PLS-DA loading plot. Dashed lines denote the center of the binned signal. Note the differences in absolute intensity.

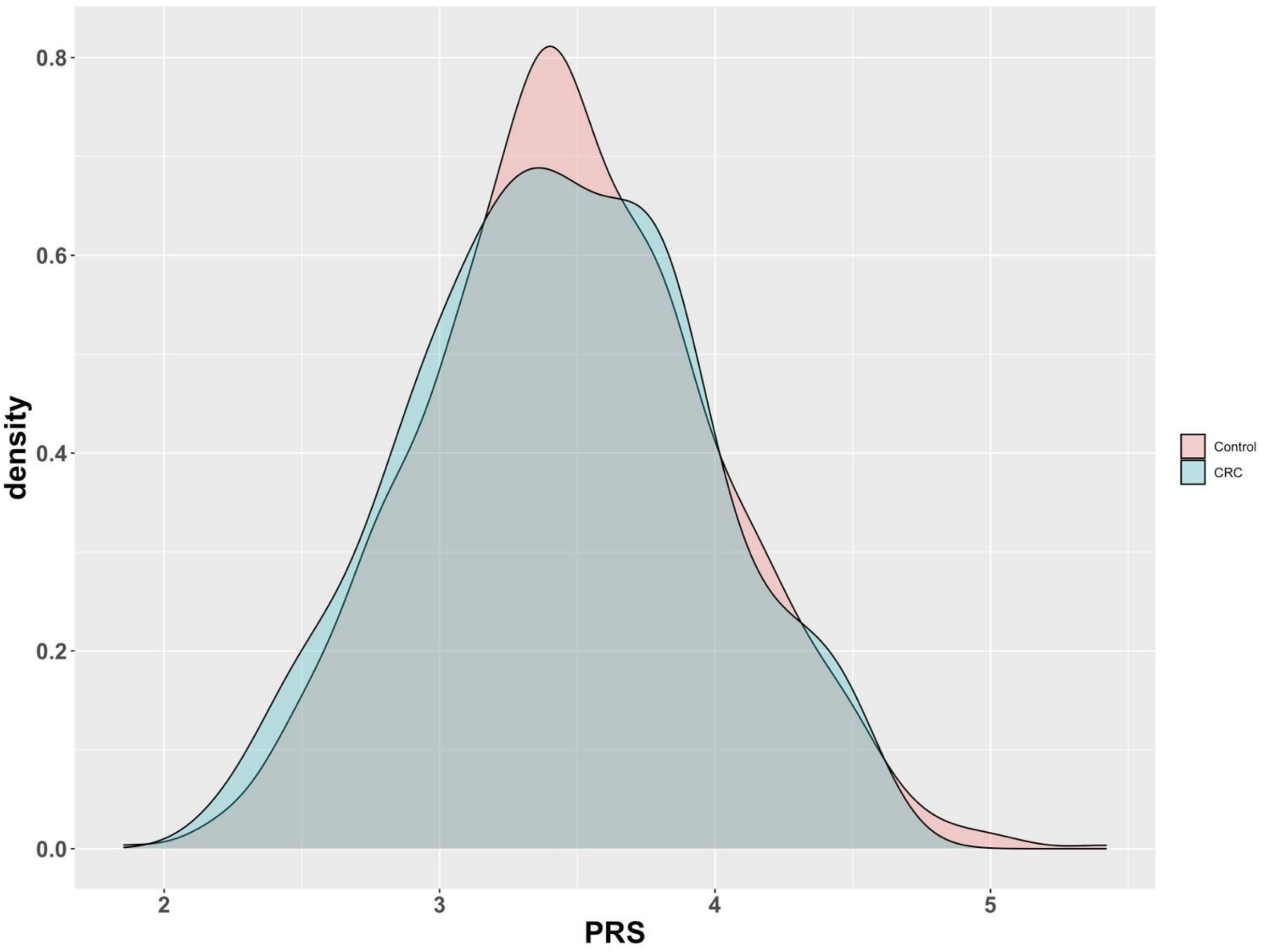

Figure S2. PRS of patients with CRC and controls based on the East Asian population.
